# Tunable Spin Injection in High-Quality Graphene with One-Dimensional Contacts

**DOI:** 10.1021/acs.nanolett.1c03625

**Published:** 2022-01-28

**Authors:** Victor H. Guarochico-Moreira, Jose L. Sambricio, Khalid Omari, Christopher R. Anderson, Denis A. Bandurin, Jesus C. Toscano-Figueroa, Noel Natera-Cordero, Kenji Watanabe, Takashi Taniguchi, Irina V. Grigorieva, Ivan J. Vera-Marun

**Affiliations:** † Department of Physics and Astronomy, University of Manchester, Manchester M13 9PL, U.K.; ‡ Facultad de Ciencias Naturales y Matemáticas, Escuela Superior Politécnica del Litoral, ESPOL, Campus Gustavo Galindo, Km. 30.5 Vía Perimetral, P.O. Box 09-01-5863, 090902 Guayaquil, Ecuador; § Consejo Nacional de Ciencia y Tecnología (CONACyT), Av. Insurgentes Sur 1582, Col. Crédito Constructor, Alcaldía Benito Juarez, C.P. 03940, Ciudad de México, México; ∥ National Institute for Materials Science, 1-1 Namiki, Tsukuba 305-0044, Japan

**Keywords:** hBN, graphene, spin injection, 1D contacts, van der Waals devices

## Abstract

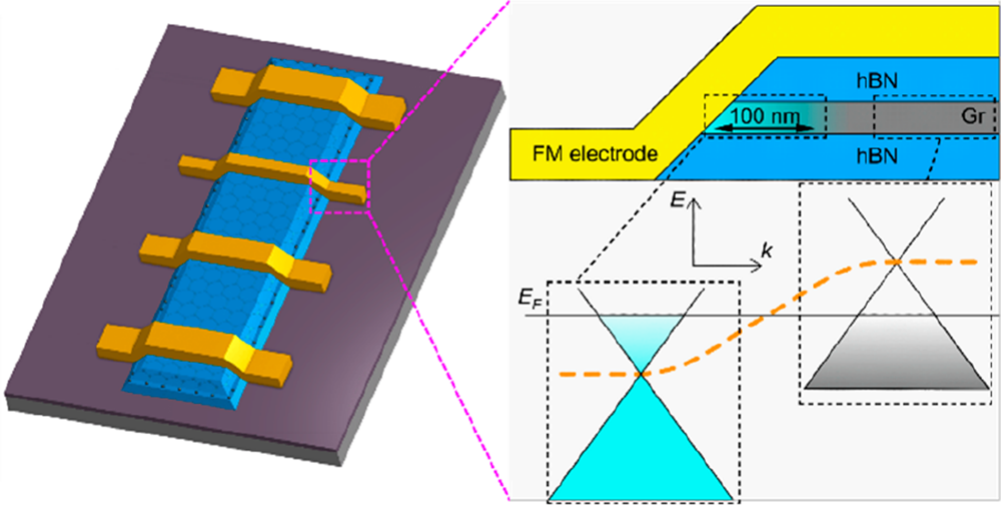

Spintronics involves the development of low-dimensional electronic systems with potential use in quantum-based computation. In graphene, there has been significant progress in improving spin transport characteristics by encapsulation and reducing impurities, but the influence of standard two-dimensional (2D) tunnel contacts, via pinholes and doping of the graphene channel, remains difficult to eliminate. Here, we report the observation of spin injection and tunable spin signal in fully encapsulated graphene, enabled by van der Waals heterostructures with one-dimensional (1D) contacts. This architecture prevents significant doping from the contacts, enabling high-quality graphene channels, currently with mobilities up to 130 000 cm^2^ V^–1^ s^–1^ and spin diffusion lengths approaching 20 μm. The nanoscale-wide 1D contacts allow spin injection both at room and at low temperature, with the latter exhibiting efficiency comparable with 2D tunnel contacts. At low temperature, the spin signals can be enhanced by as much as an order of magnitude by electrostatic gating, adding new functionality.

Graphene is currently explored for potential applications in a variety of fields due to its exceptional physical properties^1^ including high-quality electronic transport.^2,3^ In spintronics, where the spin degree of freedom is used to store, transport, and manipulate information, graphene has attracted interest as a spin transport channel.^4,5^ There has been much progress in improving spin transport characteristics in graphene^6,7^ but some challenges remain, such as the inhomogeneity in the potential profile within the channel,^8,9^ invasive tunnelling contacts,^10,11^ and impurities.^12−14^ Therefore, device architecture plays a key role to avoid the aforementioned challenges, for example via the realization of high-quality spin channels^6^ within van der Waals heterostructures.^15^ Progress in this area opens an avenue toward exploiting ballistic conduction and spin coherence in graphene-based quantum spintronics.^16−18^

Full encapsulation by hexagonal boron nitride (hBN) protects graphene from direct contact with lithographic polymers, whereas the self-cleaning process driven by van der Waals interactions limits any contamination present at the interfaces within small bubbles, ensuring atomically clean interfaces in the rest of the heterostructure.^19^ For spintronics, we need to be able to inject spin information. This is traditionally achieved via magnetic tunnel contacts. The use of tunnel barriers has grown out of the need to overcome the so-called conductivity mismatch problem,^20^ which leads to a drastically reduced spin injection efficiency when the resistance of the contact is lower than the spin resistance of the channel,^21^*R*_*s*_ = *ρλ*/*W*, with *W* the graphene channel width, λ the spin relaxation length, and ρ the graphene sheet resistance. Nevertheless, standard 2D contacts, even with the use of a tunnel barrier, are known to introduce strong doping across the channel leading to inhomogeneity^22−24^ and present challenges in the growth of the barrier without pinholes, which lead to enhanced spin relaxation.^5,25^ On the other hand, fully encapsulated graphene with 1D contacts^26^ has been shown to produce exceptionally clean devices and localize the doping within ∼100 nm near the contacts.^26−28^ 1D contacts have recently enabled spin injection in graphene,^29^ albeit only at low temperature, with graphene channel mobilities below 30 000 cm^2^ V^–1^ s^–1^ and contact polarization with multiple inversions of polarity. Therefore, further study of this architecture is warranted.

Here, we report spin transport in high-quality graphene channels with low-temperature mobility up to ∼130 000 cm^2^ V^–1^ s^–1^, fully encapsulated by hBN layers, where a spin current is injected via nanoscale-wide 1D contacts. Spin precession measurements yield a quantitative understanding by extracting the channel spin relaxation length λ and time τ_s_ and the 1D contacts’ spin injection efficiency or polarization *P*. The fabrication process produces homogeneous graphene channels, including 1D contacts that prevent substantial charge doping within the channel and offer a gate-tunable contact resistance. While the spin polarization is comparable to that from standard tunnel 2D contacts, spin transport can be electrostatically tuned into a mismatch-free spin injection regime. These elements lead to the realization of a ballistic injection process via nanoscale-wide 1D contacts and spin transport in graphene with a spin relaxation length of ∼18 μm and a long mean free path of ∼1 μm, opening up possibilities for lateral spintronic elements that exploit quantum transport.

Our devices are heterostructures consisting of monolayer graphene encapsulated between two thin (<20 nm) layers of hBN, with ferromagnetic contacts deposited directly onto narrow (∼10 nm wide) strips of graphene at the sides of the channel (see Figure 1, parts a and b). Details of device fabrication and characterization are given in the Supporting Information, Section 1. Briefly, we use the dry-peel transfer technique^30^ to prepare van der Waals heterotructures on a Si/SiO_2_ substrate. We then use standard electron beam lithography to pattern a hard polymer mask, which defines the channel geometry by using reactive ion etching.^31^ Due to different etch rates for hBN and graphene, a ∼10 nm wide step can be seen in the profile of a hBN–graphene–hBN edge (green line in Figure 1c). This step corresponds to a narrow graphene ledge,^28^ where the 1D contact is formed. For comparison, a profile of an hBN–hBN edge is shown (red line in Figure 1c) where this step is not visible. Finally, we deposit ferromagnetic contacts that pass over the channel, creating electrical connections to only the edges of the graphene layer (see inset of Figure 1a).

**Figure 1:**
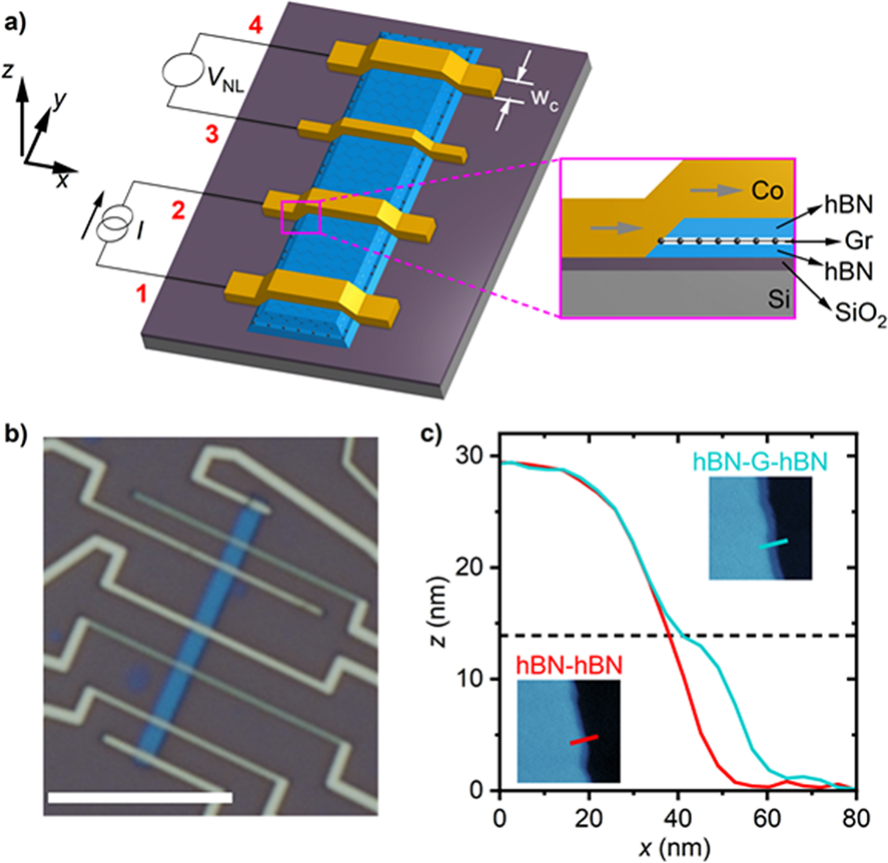
Device fabrication and characterization. (a) 3D schematic representation of an hBN–graphene–hBN channel with magnetic 1D contacts connected in a 4-probe nonlocal measurement configuration. The inset shows a cross sectional view. (b) Optical microscopy image of a typical device. Scale bar 10 μm. (c) Height profiles of a channel’s edge. Red (green) line shows the hBN–hBN (hBN–graphene–hBN) profile from the atomic force microscopy (AFM) image at the bottom-left (top-right) inset. Size of the AFM scan window is 500 nm × 500 nm. The horizontal black dotted line indicates the position where the graphene lies between the top and the bottom hBN.

Nine devices, labeled **A-I**, were studied using charge and spin transport measurements. All devices have shown qualitatively similar behavior, both at room and low temperatures (20 K). In order to characterize charge transport, we measured the four-probe resistance of graphene as a function of its charge carrier density, *n*, by using a back-gate voltage applied between the highly doped Si substrate and the graphene channel (see inset in Figure 1a). The curves in Figure 2b show the conductivity σ = 1/*ρ* of graphene at low and room temperatures for device **A**. Our devices show a uniform level of doping, within ±7 × 10^11^ cm^–2^. Given the lack of a defined doping polarity it was not possible to attribute any particular doping at the graphene edges originating from the fabrication process. To evaluate the electronic quality we extracted the corresponding field-effect mobility of the graphene channel as μ_FE_ = (dσ/d*n*)/*e*, as shown in Figure 2b, at moderate carrier densities |*n*| ∼ 1 × 10^12^ cm^–2^. For device **A**, the mobility is then 45 000 cm^2^ V^–1^ s^–1^ at room temperature and 79 000 cm^2^ V^–1^ s^–1^ at 20 K. The mobilities of our devices, typically within the range of 20 000 to 130 000 cm^2^ V^–1^ s^–1^ (see Supporting Information, Section 2), are significantly higher than previous graphene-based spintronic devices^9,10,12,32^ that exhibited mobilities <20 000 cm^2^ V^–1^ s^–1^, the majority of which have used only a partial hBN encapsulation, whereas we ensure this full hBN encapsulation throughout the spin transport channel. The charge diffusion coefficient (and corresponding mean free path) for representative devices is obtained from the graphene sheet resistance via the Einstein relation, *D* = 1/(*ρe*^2^*ν*), with ν being the density of states for single layer graphene (see Figure 2d).

**Figure 2:**
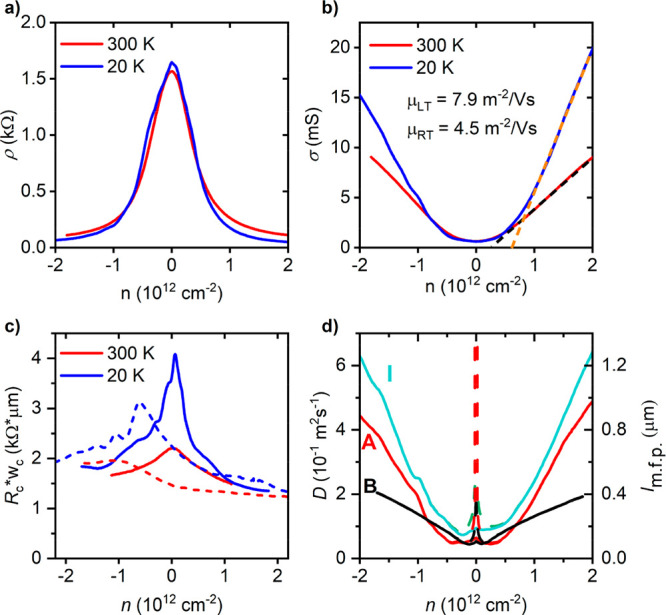
Charge transport in devices with 1D contacts. (a, b) Graphene sheet resistance (a) and conductivity (b) vs carrier density. Panel b shows the extracted field-effect mobility. Data in panels a and b are for device **A**. (c) Contact resistance-length product as a function of carrier density for two 1D contacts (continuous and dashed lines). In panels a–c, blue and red curves are for 20 and 300 K, respectively. (d) Diffusion coefficient and mean free path as a function of carrier density, at 20 K. Data in panel d correspond to three representative devices: **I** (cyan), **A** (red), and **B** (black).

To characterize the 1D contact resistance *R*_c_ as a function of carrier density *n*, as shown in Figure 2c, we use a three-probe geometry (see the Supporting Information, Section 1). The contacts exhibit typical *R*_c_ values within the range of 3–15 kΩ, which implies they should be able to inject efficiently spins into a graphene channel with *R*_s_ < 10 kΩ. Some contacts had electrical access via their two ends, see Figure 1b, which allowed a four-probe measurement to quantify the contribution of the lead series resistance (∼200 Ω, Supporting Information, Section 3). The magnetic 1D contacts present charge transport characteristics consistent with those of reported nonmagnetic ones^26^ (see the Supporting Information, Section 4). Among these are (i) an inverse scaling of *R*_c_ with the width of the contact *w*_c_ (see Figure S4a), (ii) a negligible temperature dependence of *R*_c_ for high carrier density (see Figure S4c), and (iii) a sizable dependence on carrier density, with a moderate electron–hole asymmetry and a maximum *R*_c_ near the Dirac point (see Figure 2c and Figure S4b). The electron–hole asymmetry of the contact in Figure 2c, showing a somewhat larger resistance for transport in the hole regime and a maximum *R*_c_ at *n* ≲ 0, indicates the presence of an n-doped region adjacent to the metal electrode, consistent with the difference between the work functions of the metal (Co) and graphene.^33^

Spin transport is characterized by spin-valve and spin precession (Hanle) measurements in a standard nonlocal geometry, as shown in Figure 1a. We inject a spin-polarized current *I* into graphene through contacts 1 and 2, and measure a nonlocal voltage *V*_NL_ between contacts 3 and 4. The nonlocal resistance is defined as *R*_NL_ = *V*_NL_/*I*. The spin valve signal is given by the difference between the two distinct levels corresponding to the parallel and antiparallel alignment of the injector and detector electrodes, Δ*R*_NL_ = *R*_NL_^P^ – *R*_NL_^AP^. Spin signals were measured for different separations between injector and detector, *L*, ranging from 2 to 15 μm (see the Supporting Information, Section 7). An increase of approximately 1 order of magnitude in Δ*R*_NL_ from room to low temperature was observed, as shown in parts a and b of Figure 3, with the latter reaching Δ*R*_NL_ > 1 Ω. This strong temperature dependence is distinct from the weaker dependence observed in standard tunnel 2D contacts,^5^ indicating a different transport mechanism for spin injection in 1D contacts.

**Figure 3:**
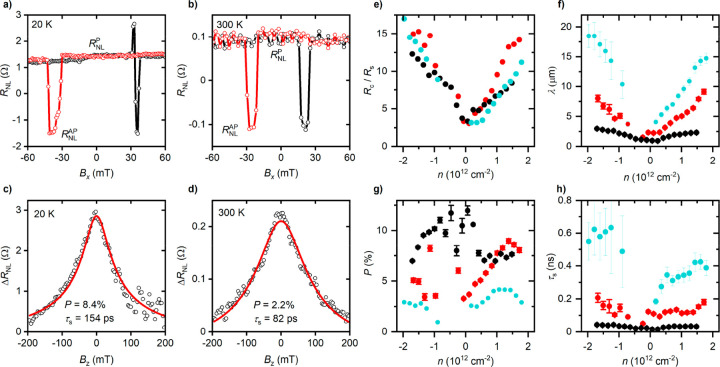
Spin transport in devices with 1D contacts. (a, b) Spin valve measurements. The black (red) curve represents the up (down) sweep of in-plane magnetic field. (c, d) Hanle spin precession measurements. The red curve is a fit to the Bloch equation, using the parameters shown in the panel. Data in panels a–d are for device **A**, with *L* = 2.4 μm. Panels a and c (b and d) are for low (room) temperature. (e–h) Spin transport parameters. Contact resistance to channel spin resistance ratio (e), spin relaxation length (f), spin polarization (g), and spin relaxation time (h) as a function of carrier density, at 20 K. Data in panels e–h correspond to the same three representative devices as in Figure 2d: **I** (cyan), **A** (red), and **B** (black).

The observation of a Hanle signal^34^ enables us to confirm the presence of spin transport and rule out spurious contributions which hindered previous efforts in devices with 1D contacts.^32^ Here *R*_NL_^P^ and *R*_NL_^AP^ are measured while sweeping an external magnetic field applied perpendicular to the device plane (*B*_*z*_). This causes the injected spins, having a polarization along the *x*-direction (see Figure 1a), to precess within the *x*–*y* plane while moving within the channel. The resulting Δ*R*_NL_(*B*_*z*_) (see parts c and d of Figure 3) is analyzed using a solution to the steady-state Bloch equation^34,35^
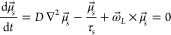
1where *μ⃗*_*S*_ is the spin accumulation within the channel and *D* is the diffusion coefficient, the latter being equal to the charge diffusion coefficient in the absence of spin Coulomb drag.^36^ The term *ω⃗*_*L*_ × *μ⃗*_*S*_ describes spin precession under an external magnetic field *B⃗*, where the Larmor frequency is given by *ω⃗*_*L*_ = g *μ*_*B*_/*ℏ B⃗*, with the Lande factor g = 2 and *μ*_*B*_ the Bohr magneton. Therefore, the spin transport parameters (τ_s_, *D* and ) of the channel are extracted.

The spin signal Δ*R*_NL_ depends on the spin polarization of the magnetic 1D contacts, *P*. In the absence of spin precession (*B*_*z*_ = 0), the spin signal is described by a balance of spin injection and relaxation within the channel, given by^34^
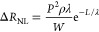
2with *L* the injector–detector distance and *W* the channel width. The channel parameters for charge (σ ∝ *D*) and spin (τ_s_) transport have a moderate 2-fold increase at low temperature, see Figures 2 and 3. A significant increase in conductivity at low temperature, in the high-density regime, is expected in high-quality graphene due to the absence of acoustic-phonon scattering.^26^ Furthermore, *P* has a 4-fold increase at low temperature, as confirmed by the parameters extracted from Hanle measurements. These observations indicate that the strong temperature dependence seen in Δ*R*_NL_ is dominated by the polarization of the contacts, via the scaling Δ*R*_NL_ ∝ *P*^2^.

The identification of an n-doped graphene region next to our 1D contacts from the electron–hole asymmetric response of *R*_c_, together with the observation of a more electron–hole symmetric channel resistance, as seen in Figure 2a, can be understood by considering the distinct geometries and length scales involved in both measurements. For the case of the contact resistance, the direct metal–graphene contact occurs only within the 1D edge, identified as the <10 nm step in the heterostructure profile; see Figure 1c. Furthermore, the potential profile from the n-doped graphene region in direct contact with the metal is expected to extend to just ∼100 nm^37,38^ to the rest of the channel. This doped region only exists near the 1D contact, which in our devices has a nominal width *w*_c_ of 100—350 nm. The n-doped region being localized in the vicinity of the 1D edge is in stark contrast to standard 2D junctions which cover the full width of the channel and lead to substantial inhomogeneity.^22−24^ Here, the geometry of the 1D contacts plays a key role. On the other hand, for the case of the channel sheet resistance, most of the channel is undoped, except for the nanoscale region in direct vicinity of the 1D edge. Therefore, the channel resistance, probed at a length scale *L*, *W* ≥ 1 μm, exhibits a more electron–hole symmetric response.

A further characteristic that distinguishes these magnetic 1D contacts with nanoscale geometry from the behavior in standard 2D junctions^33,38^ and is hitherto unaddressed in nonmagnetic^26,28^ or magnetic^29,32^ 1D contacts, is a nonmetallic increase in resistance at low temperature. All measured 1D contacts (see Figure 2c) consistently exhibit a marked carrier density-dependent contact resistance at low temperature, indicating that *R*_c_ varies with the Fermi energy. This dependence is considerably weaker at room temperature. At low carrier density, *R*_c_ typically exhibits up to a 2-fold increase at low temperature. This behavior is distinct from that of both the graphene channel sheet resistance, which is essentially temperature-independent (see Figure 2(a)), and the typical 2D metal–graphene ohmic junctions.^33,38^ Only at high carrier density is this temperature dependence of *R*_c_ reduced, consistent with the weak temperature dependence observed in 1D contacts with^26^*w*_c_ > 1 μm.

The description of contact resistance in 2D metal–graphene junctions involves two processes: carrier transport from the metal to the (doped) graphene region in direct contact with the metal and transport from the doped graphene region to the rest of the (undoped) graphene channel. The transmission for those two processes gives rise to the observed contact resistance.^38^ The first process is constrained by the number of conduction modes in the n-doped graphene region, which is limited by the contact width. Within this description, our nanoscale contacts limit the number of conduction modes in the junction and reduce the conductance at the metal–graphene interface, so that ballistic transport across a width of ∼100 nm at low temperature would still lead to a sizable *R*_c_.^28,38^ The latter enables spin injection, which in graphene has traditionally required the use of tunnel barriers^20^ in order to overcome the conductivity mismatch problem^21^ that arises when the injected spins are backscattered into the magnetic contact where they rapidly relax their spin orientation. In this case, the linear room temperature response corresponds to the thermally smeared Sharvin resistance.^39^ With regards to the second process, transport occurs across a potential profile in graphene of ∼100 nm length scale, smaller than the mean free path in most of our channels. Crucially, this energy-dependent process is tunable in 1D junctions since, unlike 2D junctions where the Fermi level of graphene under the contacts is strongly pinned, the Fermi level of graphene near a 1D junction can be tuned efficiently,^29^ resulting in a tunable contact resistance. This difference between 2D junctions and 1D junctions derives from their distinct dimensionality and scale of the metal–graphene interface (<10 nm) and the doped graphene region (∼100 nm); see Figure 4b.

**Figure 4:**
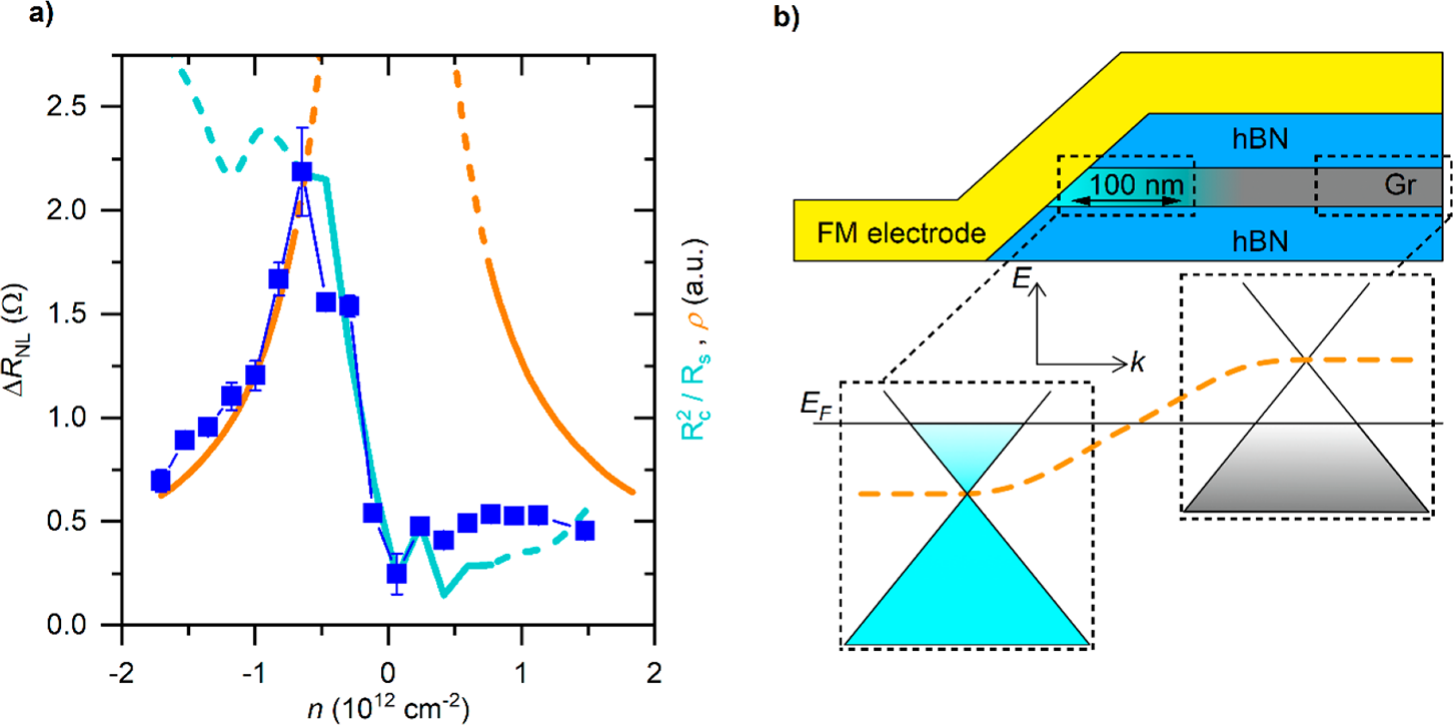
Tunable spin injection efficiency at 20 K. (a) Spin valve signal as a function of carrier density for device **C** (blue squares) with *L* = 5.6 μm. The orange (cyan) line represents the graphene sheet resistance (contact to spin resistance ratio), with each line turning solid to indicate a similar scaling as the spin valve signal. (b) Schematic representation of a magnetic 1D contact to an hBN–graphene–hBN channel, with the orange dashed line depicting the position of the neutrality point within graphene.

Parts f–h of Figure 3 show spin transport parameters extracted from Hanle measurements, for three representative devices at 20 K. Device **I** (cyan data) has a high electronic quality close to the upper limit of our mobility range, with μ_FE_ = 130 000 cm^2^ V^–1^ s^–1^. Device **A** (red data) also has a high electronic quality, with μ_FE_ = 79 000 cm^2^ V^–1^ s^–1^, whereas device **B** (black data) has a lower quality (μ_FE_ = 27 000 cm^2^ V^–1^ s^–1^). As shown in Figure 3g, we achieve contact polarization values of up to ∼10%, comparable with standard tunnel contacts on graphene^25,34^ (see also Figure S5c).

The spin relaxation time τ_s_ and length λ, shown in parts f and h of Figure 3, describe the spin transport within the graphene channel. Both parameters exhibit an approximate electron–hole symmetry, consistent with the graphene sheet resistance (see Figure 2a). Moreover, their magnitude correlates with the electronic quality, with τ_s_ and λ for device **I** being about twice as large as those for device **A**, while the parameters for device **A** are ca. three times as great as for device **B**. This observation indicates spin relaxation dominated by the Elliot–Yafet mechanism,^40,41^ where τ_s_ increases with the diffusion coefficient *D* (see Figure 2d). A priori, we cannot rule out contributions from other mechanisms, as previous studies of graphene–hBN heterostructures have found contributions from both Elliot–Yafet and D’Yakonov–Perel mechanisms,^42^ and there might also be other sources of spin relaxation due to the direct ferromagnetic contacts to graphene.^43^ To ascertain that spin absorption at the ferromagnet–graphene interfaces did not play a significant role, we used the extended expression for Δ*R*_NL_ that included contact resistances.^35^ The corresponding fit is shown in Figure S7 (Supporting Information). It is clear that, with our experimental accuracy, it is indistinguishable from the fit obtained using eq 1, and that the extracted values of τ_s_ are not significantly affected. Further indication for the role of the Elliot–Yafet mechanism is obtained by studying the carrier density dependence. For all our devices, τ_s_ and λ increase with increasing carrier concentration, having a minimum close to the Dirac point. This behavior, also observed in single layer graphene using 2D contacts, has been attributed to the Elliot–Yafet mechanism^44,45^ and results in a linear scaling between spin relaxation time τ_s_ and momentum relaxation time *τ*_*p*_ (see the Supporting Information, Section 6). We have observed spin transport across distances of 15 μm, while crossing several (noninvasive) 1D contacts, only limited by device dimensions (see Figure S7). Overall, the demonstration of spin transport parameters reaching values up to *D* ∼ 0.7 m^2^ s^–1^ and λ ∼ 18 μm opens up the exploration of lateral spintronic architectures involving long distance spin transport,^42^ now within high-quality and homogeneous channels.

We note that, despite the clear indications that Elliot–Yafet mechanism of spin relaxation plays a dominant role in our devices, the observed τ_s_ is rather short, in fact shorter than in many devices with 2D contacts and of lower quality reported in the literature.^10,12^ In the latter case, τ_s_ ∼ 2 ns or more have been observed, while the maximum spin lifetime in our device **I** with the highest mobility is only ∼0.7 ns. We speculate that the reason is likely to be due to the specific geometry of spin injection through 1D contacts: here spin-polarized electrons enter from the opposite sides of a graphene channel which is followed by propagation in the perpendicular direction along the channel. In this case, spin transport becomes essentially 2D and may not be accurately described by the standard 1D Hanle model (where spins are injected uniformly across the channel and continue propagation in the same direction).

We evaluate the degree of conductivity mismatch via the ratio of contact resistance, *R*_c_, to spin resistance of the graphene channel, *R*_s_, where a ratio *R*_c_/*R*_s_ ≫ 1 indicates a regime of efficient spin injection.^5,11^ In graphene spintronic devices, the ratio *R*_c_/*R*_s_ has been tuned by using an electrostatic back-gate via the carrier density dependence of ρ and λ, whereas for tunnel junctions *R*_c_ typically exhibits a weak dependence.^5,25^ In our work, not only the channel resistance but also the 1D contacts exhibit a dependence on carrier density (see Figure 2c), which, as we show below, can be used to overcome the impedance mismatch problem. As shown in Figure 3e, the ratio remains *R*_c_/*R*_s_ > 1 for all carrier densities, increases with carrier density, and it is tunable to values *R*_c_/*R*_s_ ≫ 1 at high density.

To further evaluate the spin injection efficiency and its tunability, we discuss the spin valve signal, Δ*R*_NL_. As shown in Figure 4a, Δ*R*_NL_ has a minimum at the Dirac point, consistent with the minimum observed for the spin transport parameters (Figure 3, parts f and h). Away from the Dirac point, Δ*R*_NL_ exhibits a nonmonotonic behavior: in the hole regime, the signal increases and reaches a maximum for *n* ∼ −0.8 × 10^12^ cm^–2^, after which it decreases. For the electron regime, this behavior is much less pronounced and the spin signal remains comparatively low. This overall behavior is understood by considering two distinct regimes of conductivity matching.^25^ First, at high carrier density, where *R*_c_/*R*_s_ ∼ 10, there is a regime free of conductivity mismatch. Here, Δ*R*_NL_ is insensitive to the value of *R*_c_, and it scales as Δ*R*_NL_ ∝ ρ (see eq 2). This is observed in the hole regime, as indicated by the orange curve in Figure 4a. On the other hand, at low carrier density, *R*_c_/*R*_s_ ∼ 3; see Figure 3e. The latter corresponds to an intermediate regime where, although nominally in a conductivity-matched condition, the spin transport is still sensitive to the value of *R*_c_. In this case Δ*R*_NL_ exhibits a scaling similar to that in the mismatch regime^46^ ∝ *R*_c_^2^/*R*_s_. As indicated by the cyan line in Figure 4a, the latter regime accounts for the reduction in Δ*R*_NL_^5,11^ at low carrier density and in the electron regime.

This device architecture demonstrates efficient spin injection in graphene using nanoscale-wide 1D contacts, reproducibly across several devices. The ballistic spin injection process allows for observation of the Hanle effect^32^ and tunable spin signal and achieves a mismatch-free regime at moderate carrier density. The spin signal shows a consistent behavior across all devices, with a minimum near the Dirac point. This observation is compatible with the proposal of magnetic proximity at the Co–graphene interface,^29,33^ where a reversal in the polarity of the spin polarization is expected near the Dirac point, implying no spin polarization and thus no spin signal near the Dirac point. The fact that our devices do not show any inversion of the spin signal as a function of carrier density is consistent with a homogeneous potential profile within the graphene channel, where both injector and detector contacts would reverse their polarizations at similar carrier density. The demonstration of spin transport in graphene with a large mean free path of ∼1 μm at low temperature, comparable to device dimensions, while ensuring sizable spin relaxation lengths, is a key advance in the development of low-dimensional spintronic systems approaching^47^ the high electronic mobility of state of the art charge-based devices.^26,48^

## Supporting Information Available

Details on the overall device fabrication and characterization, characterization of additional devices, contributions to the contact resistance, behavior of the one-dimensional magnetic contacts, an additional device with analysis of Hanle spin precession, exploration of the mechanism of spin relaxation, and long distance spin transport (PDF)
