# Supplementary material for: Tunable Spin Injection in High-Quality Graphene with One-Dimensional Contacts
Source: Nano Lett. 2022 Jan 28;22(3):935–41. doi: 10.1021/acs.nanolett.1c03625 (PMC9098166; doi:10.1021/acs.nanolett.1c03625)
Supplement: Supplementary file 1 — nl1c03625_si_001.pdf [file nl1c03625_si_001.pdf]

SUPPORTING INFORMATION

Tuneable spin injection in high-quality graphene with one-dimensional contacts

Victor H. Guarochico-Moreira,[1,2] Jose L. Sambricio,[1] Khalid Omari,[1] Christopher R. Anderson,[1] Denis A. Bandurin,[1] Jesus C. Toscano-Figueroa,[1,3] Noel Natera-Cordero,[1,3] Kenji Watanabe,[4] Takashi Taniguchi,[4] Irina V. Grigorieva,[1*] and Ivan J. Vera-Marun[1*]

[1]*Department of Physics and Astronomy, University of Manchester, Manchester M13 9PL, United Kingdom*

[2] *Facultad de Ciencias Naturales y Matemáticas, Escuela Superior Politécnica del Litoral, ESPOL, Campus Gustavo Galindo Km. 30.5 Vía Perimetral, P.O. Box 09-01-5863, 090902, Guayaquil, Ecuador*

[3]*Consejo Nacional de Ciencia y Tecnología (CONACyT), Av. Insurgentes Sur 1582, Col. Crédito Constructor, Alcaldía Benito Juarez, C.P. 03940, Ciudad de México, México*

[4]*National Institute for Materials Science, 1-1 Namiki, Tsukuba 305-0044, Japan*

Corresponding Authors

*E-mail: irina.grigorieva@manchester.ac.uk

*E-mail: ivan.veramarun@manchester.ac.uk

Section 1. Device fabrication and characterisation

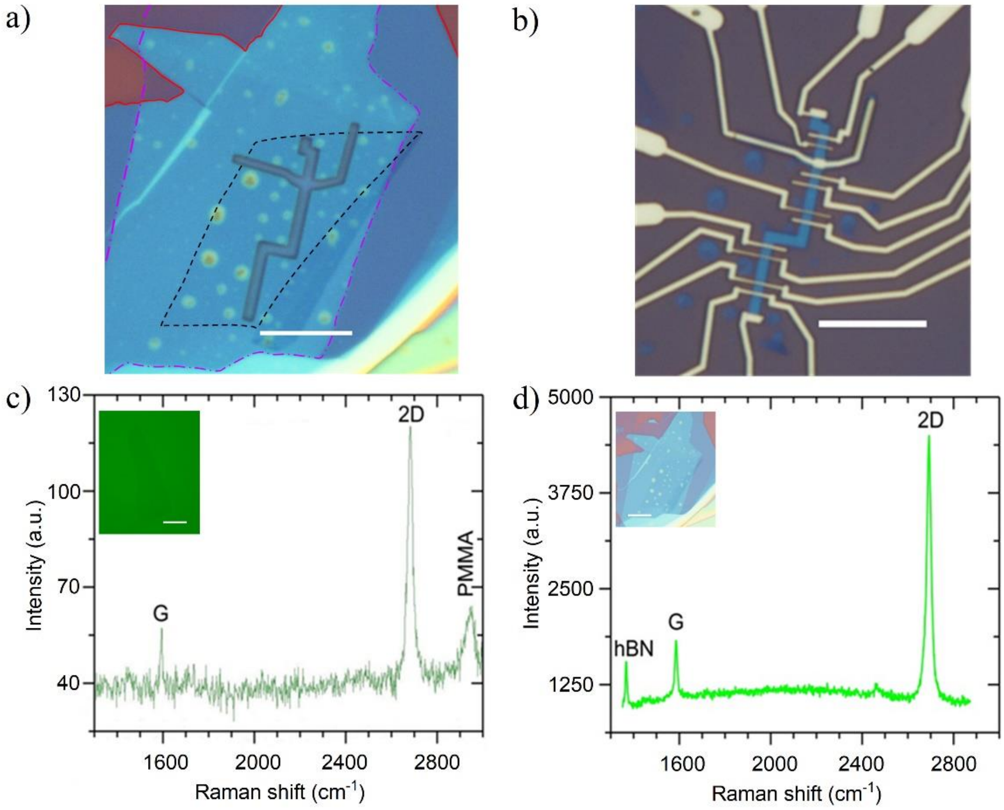

Figure S1.- Device fabrication and Raman characterisation. a, Optical microscopy picture of our hBN-graphene-hBN stack with a PMMA mask on top to define the encapsulated graphene channel. Solid red, dot-dashed purple and dashed black lines represent the boundaries of the top hBN, bottom hBN and graphene, respectively. **b,** Optical microscopy picture of one typical device, after depositing the magnetic contacts. **c,** Raman spectrum of graphene on a PMMA/PMGI stack. Inset shows a green-filter optical image of the graphene flake in the stack in (**a**). **d,** Raman spectrum of the hBN-graphene-hBN heterostructure. Inset shows an optical image of the stack in (**a**). All scale bars are 10 µm.

The hBN-graphene-hBN heterostructures were assembled on a highly p-doped Si substrate, with a SiO_2 layer of 290 nm thickness. Figure S1a depicts an optical image of a typical hBN-graphene-hBN stack assembled using the dry-peel transfer technique[1]. Graphene and hBN were mechanically exfoliated on top of a polymer stack consisting of poly(methylglutarimide) (PMGI) and poly(methyl methacrylate) (PMMA), previously coated onto a Si substrate. The sacrificial PMGI layer is dissolved with a water-based solvent (MF319) while the top layer together with the desired hBN flake is lifted off from the Si substrate. The resulting PMMA membrane is placed onto a metal ring and loaded face down into a setup with micromanipulators to align the top hBN with the graphene flake, previously prepared on the polymer stack. The graphene flake is picked up by the top hBN flake attached to the PMMA membrane, via the dry-peel transfer technique[1]. This structure is aligned with a second (bottom) hBN flake, previously exfoliated on a Si substrate, to finalise the assembly of the heterostructure. The PMMA membrane is dissolved in acetone, followed by annealing at 300°C in an atmosphere of Ar/H_2 gas mixture.

A self-cleaning process[2] coerces any contamination (hydrocarbons and absorbed water), present at the surfaces of graphene and hBN, to cluster into submicron-sized bubbles during assembly. This ensures atomically clean interfaces in the majority of the heterostructure. The bubbles are identified by optical and atomic force microscopy and thus can be avoided when defining the channel's geometry (figure S1a). Electron beam lithography (EBL) is used to pattern a PMMA hard mask to define the channel, which is created using reactive ion etching with a mixture of CHF_3 and O_2 gases[3]. Next, we used a second step of EBL to pattern the contacts in a PMMA resist, followed by deposition of Co by electron-beam evaporation under a base pressure vacuum of 10^{-6} mbar, as shown in figure S1b.

The characterisation of our devices begins with confirmation that a single layer of graphene has been isolated. This is done using Raman spectroscopy, either following the exfoliation of graphite on PMMA (figure S1c) or following the assembly of the stack (figure S1d). Both spectra show the G and 2D peaks characteristic of single layer graphene (at ~1580 and ~2700 cm^{-1} respectively). Additionally we observe peaks characteristic of PMMA or hBN (at ~1340 and ~2950 cm^{-1} respectively).

For electrical characterisation, all devices were measured in a cryostat, under a vacuum atmosphere of pressure < 6 x 10^{-7} mbar, using standard lock-in techniques at low-frequency (< 20 Hz). To electrostatically gate the graphene and to bias the contacts we used dc sources. The gate voltage, V_{bg}, induces a charge carrier density $n = C_{\mathrm{g}}(V_{\mathrm{bg}} - V_{\mathrm{D}})/e$, where e is the elementary charge, C_{g} is the geometrical gate capacitance per area, and V_{D} is the gate voltage for the maximum of ρ. We determine C_{g} by considering two dielectrics in series, the SiO_2 and the bottom hBN, the latter with a thickness determined by AFM. We extract the field-effect mobility of the graphene channel as $\mu_{\mathrm{FE}} = (d\sigma/dn)/e$, with $\sigma = 1/\rho$. The diffusion coefficient is extracted from the Einstein relation, $D = 1/(\rho e^2 \nu)$, by using the density of states for single layer graphene, $\nu = \sqrt{\mathrm{g_s g_v} n}/\sqrt{\pi}\hbar v_{\mathrm{F}}$, where $\mathrm{g_s}$ and $\mathrm{g_v}$ are the spin and valley degeneracy respectively, and $v_{\mathrm{F}} = 10^6$ m/s the Fermi velocity. The mean free path is given by $l_{\mathrm{m.f.p.}} = 2D/v_{\mathrm{F}}$, as shown in Fig. 2d.

Following the notation in Fig. 1a, to measure the R_{c} of contact 2 in a three-probe geometry, current is applied via contacts 2 and 1 while voltage is measured between contacts 2 and 3. With this geometry we measure contributions of the 1D contact junction resistance, the spread resistance within the graphene channel, and the series lead resistance. The first contribution dominates R_{c} and is discussed in the main text. Both the second contribution, due to the detection of charge current (Maxwell) spreading resistance[4,5], and the third contribution, due to the metallic electrode and measurement setup lead, are found to be negligible (see Supporting Information Section 3). For the evaluation of conductivity mismatch we use the R_{c} values of both the injector and detector electrodes[6].

For the non-local geometry, the electrical connections are schematically shown in Fig. 1a, as described in the main text. Contacts 1 and 4 are generally chosen to be at the ends of the channel, so that spin transport is dominated only by contacts 2 (injector) and 3 (detector). A magnetic field is applied along the length of the magnetic electrodes (in-plane B_x) to configure

the injector and detector electrodes in a parallel or an antiparallel state, corresponding to two distinct levels of the non-local resistance, R_{NL}^{P} and R_{NL}^{AP} (see Fig. 3a and Fig. 3b). This geometry separates the spin current from the (drift) charge current, reducing spurious magnetoresistance effects.

Section 2. Characterisation of additional devices

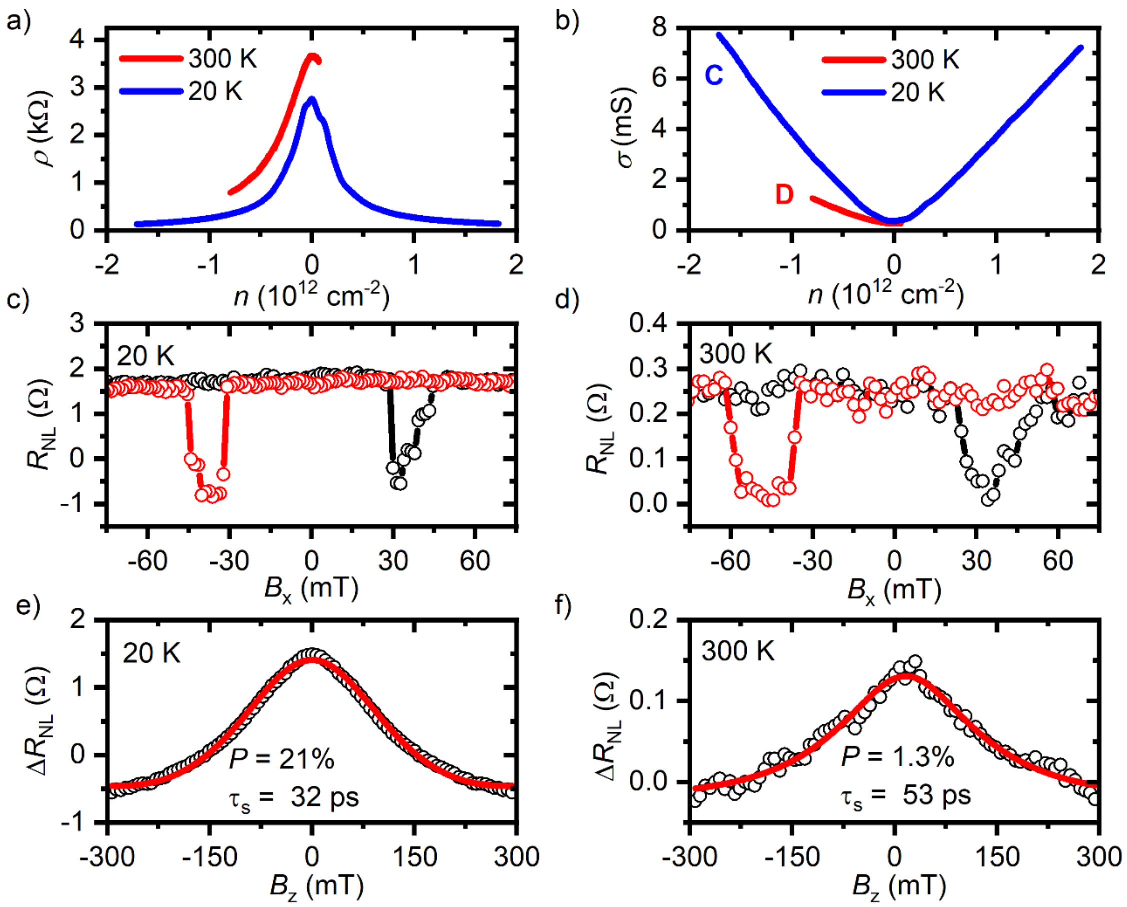

Figure S2. Electrical Characterisation for device C (at 20 K) and D (at 300 K). a, Dependency of graphene resistivity on charge carrier density. **b,** Dependency of conductivity on charge carrier density. **c, d,** Spin valve measurements at $n \sim -0.5\text{x}10^{12}$ cm^{-2}. The black (red) curve is for the up (down) sweep of the in-plane magnetic field. **e, f,** Hanle spin precession curves at $n \sim -0.5\text{x}10^{12}$ cm^{-2}. The solid lines are fits to the one-dimensional Bloch equation.

In this section we present data from the electrical characterisation of additional devices. Figure S2 shows the charge and spin transport of device C at 20 K and device D at 300 K. Device C has a distance between injector and detector of 5.6 µm and device D a distance of 1.5 µm. We typically observe the magnitude of the spin signal to be in the range of few hundreds of milliohms at 300 K, whereas at 20 K these values are one order of magnitude greater. This scaling appears to be dominated by the polarization of the magnetic contacts rather than by the spin relaxation in the channel (discussed in the main text and later here in Section 4).

A further summary of device geometry, layer thicknesses, and charge transport parameters for all devices is presented in table S1. As we can see, the mobility in our devices spans a range

from ~ 10,000 $cm^2V^{-1}s^{-1}$ to ~ 50,000 $cm^2V^{-1}s^{-1}$ at 300 K. Whereas at 20 K this range spans from ~ 20,000 $cm^2V^{-1}s^{-1}$ to ~ 130,000 $cm^2V^{-1}s^{-1}$ (see table S1). We attribute these variations to the quality of edges, small bubbles and effects of strain. The last two have been greatly avoided thanks to the progressive improvement of the stacking process[7].

**Device**	**b-hBN (nm)**	**t-hBN (nm)**	***L* (μm)**	***W* (μm)**	**μ_{FE} RT ($cm^{-2}V^{-1}s^{-1}$)**	**μ_{FE} LT ($cm^{-2}V^{-1}s^{-1}$)**	**n^* (cm^{-2})**	***D* (m^2s^{-1})**	**$l_{m.f.p.}$ (μm)**
A	6	9	2.4	1.1	45,000	79,000	$3.5x10^{11}$	0.47	0.93
B	12	15	5.1	1.2	19,000	27,000	$1.5x10^{11}$	0.20	0.40
C	12	15	5.6	1.2	19,000	30,000	$1.4x10^{11}$	0.20	0.41
D	10	12	1.5	1	16,000	17,000	$3x10^{10}$	0.06	0.12
E	5	15	2.6	1.2	12,000	14,000	$4x10^{10}$	0.06	0.13
F	7	14	1	1	12,000	16,000	$1.3x10^{11}$	0.07	0.14
G	12	15	12.7	1.2	18,000	28,000	$1.6x10^{11}$	0.20	0.39
H	6	9	3.1	2.4	41,000	85,000	$3.3x10^{11}$	0.52	1.05
I	6	9	3.1	2.4	47,000	130,000	$3.3x10^{11}$	0.64	1.3

Table S1.- Summary of device parameters. The field-effect mobility, μ_{FE}, is extracted for most devices at $|n|$ ~ 1—$1.5x10^{12}$ cm^{-2}, with the exception of devices **D** and **E** where this is done at n = -$0.5x10^{12}$ cm^{-2}. The residual carrier density, n^*, charge diffusion coefficient, D, and the mean free path, $l_{m.f.p.}$, are extracted at 20 K. For most devices D and $l_{m.f.p}$ are extracted at $|n|$ = $2x10^{12}$ cm^{-2}, their maximum values, except for **D** and **E** where these parameters are extracted at n = -$0.5x10^{12}$ cm^{-2}.

All the devices included in Table S1 demonstrated spin transport. Their characteristics were generally in line with our results discussed in the main text. In particular, they exhibited a strong temperature dependence, with spin-valve signal on the order of ohms at 20 K and a decrease by about one order of magnitude at room temperature. Table S2 presents a summary of the non-local spin valve signals observed both at 20 K and at room temperature.

**Device**	**ΔR_{NL} @ 20 K (Ω)**	**ΔR_{NL} @ RT (Ω)**
A	2.7	0.2
B	0.62	—
C	2.4	—
D	—	0.2
E	0.29	0.03
F	2.0	—
G	0.75	—
H	1.0	0.23
I	1.3	0.09

Table S2.- Summary of spin-valve response. Representative values of the spin valve signal, ΔR_{NL}, observed for each device, for both 20 K and room temperature (RT). Cases where no measurement took place at a certain temperature are labelled '—' .

Section 3. Contributions to the contact resistance R_C

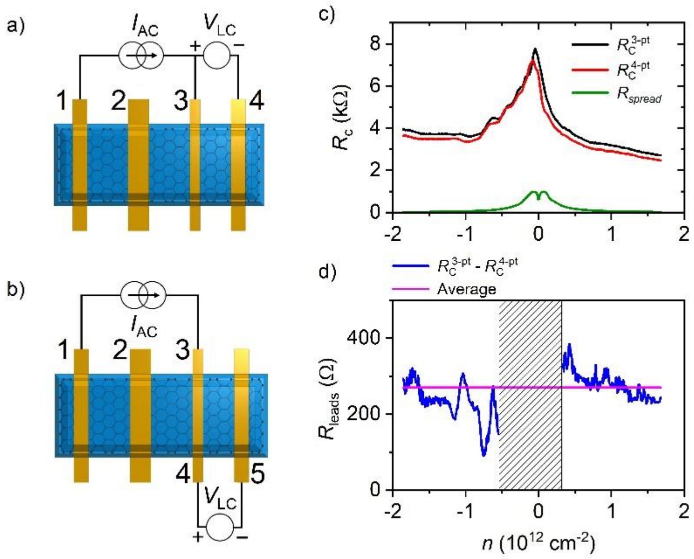

Figure S3. Electrical characterisation of contact resistance. a, b, Schematic of a 3-point (a) and 4-point (b) measurement configuration of R_C. **c,** Contact resistance as a function of carrier density for a typical contact, designed to perform measurements with both configurations (black and red curves respectively). Green curve is the calculated Maxwell spread resistance using the resistivity of the graphene channel. **d,** Estimation of the lead resistances by subtracting the 3-point and 4-point measurements.

We use both a 3-point method and a 4-point method, schematically represented in figure S3a and b respectively, to extract the contribution to the contact resistance coming from the leads connected to the contacts. Figure S3c shows the resistance of a typical contact measured with these two methods (black and red), as a function of carrier density. When both measurements are subtracted, we obtain a gate-independent value of around 250 Ω shown in figure S3d. We have found that this series resistance contribution is in the range of 100 to 300 Ω, for all our contacts. Therefore a value of 200 Ω has been deducted from the 3-point raw data for all devices. The shaded area in figure S3d indicates the range of carrier density where the deduction becomes unreliable due to variations in the junction resistance at low carrier density.

We analyse another possible contribution to the contact resistance, originating from charge current spreading, known as the Maxwell spreading resistance. We use the following approximation[5] for our device geometry,

$$R_{\text{spread}} = \frac{\rho}{2\,\pi} \ln\left(\frac{W}{l_{\text{m.f.p.}}}\right), \tag{1}$$

where ρ is the resistivity of graphene, W the width of the channel and $l_{\text{m.f.p.}}$ is the mean free path. The green curve in figure S3c shows the results of applying equation 1 to the measured resistivity as a function of carrier density. It is clear that its value is much lower than the contact resistance, demonstrating that the contribution of this term does not dominate the measured value of R_C.

Section 4. Behaviour of one-dimensional magnetic contacts

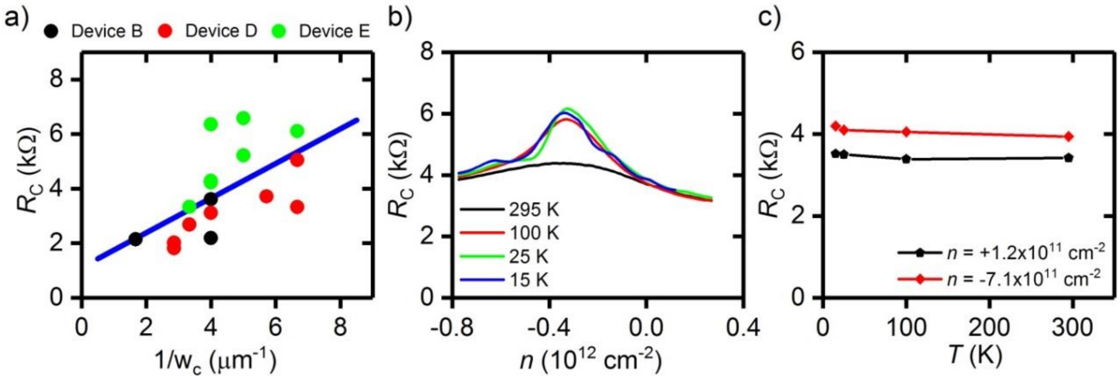

Figure S4. 1D magnetic contacts. a, Contact resistance scaling with the inverse of the contact width. R_C is taken at 20 K and at moderate carrier density $n - n_0 \approx + 1.5x10^{12}$ cm^{-2}, where n_0 is the carrier density at the R_C maxima. The blue line is a global linear fit of the data. **b,** Contact resistance as a function of carrier density, for different temperatures. **c,** Contact resistance as a function of temperature at two fixed carrier densities, for the same data as in **b**.

The width of the 1D contacts (w_c) was varied from 100 to 350 nm, to ensure different contacts reorient their magnetisation at different values of the field applied along their easy axis, B_x (see Fig. 1(a)), for the purpose of spin valve measurements. This also enabled the possibility to characterise the scaling of their resistance with said widths. Figure S4a shows the scaling of contact resistance with the inverse of its width, measured on three different devices. The approximate scaling $R_c \propto 1/w_c$ is consistent with previous reports[8].

Figure S4b shows the dependence of the contact resistance with carrier density at different temperature, for a representative contact. The peak seen in all our contacts appears at $n \lesssim 0$, consistent with previous reports on the n-type doping of graphene in 2D Co contacts[9,10]. We have seen a significant increase in this peak resistance at low temperature, as depicted in Figure S4b. This is attributed to the emergence of ballistic transport at low temperature. At high carrier density, the contact resistance shows a negligible dependence on temperature (see Figure S4c), in agreement with the reported behaviour of 1D contacts fabricated with different materials[8]. Note the resistance of 1D contacts is material dependent. In the case of cobalt we found the contact resistance-width product to be in the range of 1 – 5 kΩ*μm.

Section 5. Additional device with analysis of Hanle spin precession

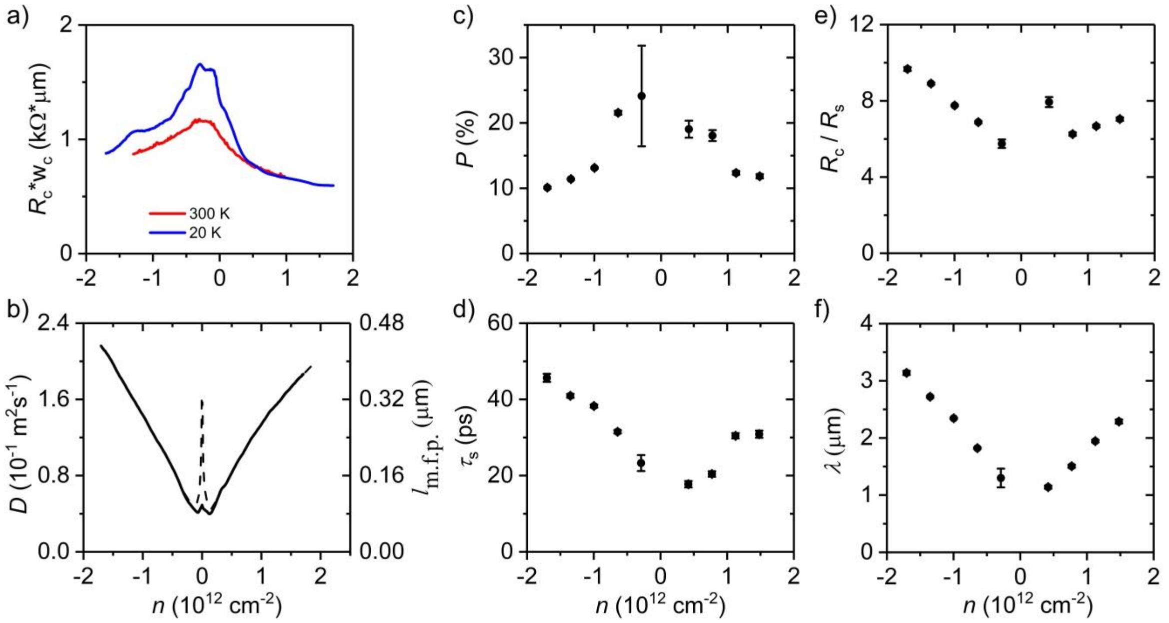

Figure S5. Extraction of spin transport parameters from device C. **a,** Contact resistance length product as a function of carrier density for a typical contact. **b,** Charge diffusion coefficient D as a function of charge carrier density at 20 K. D was calculated from the Einstein relation for an ideal DOS (dashed line) and for a DOS with a Gaussian broadening of ~40 meV (solid line). The mean free path (right axis) is extracted from the relation $l_{\text{m.f.p.}} = 2D/v_{\text{F}}$. **c, d, e, f,** Spin polarisation (**c**), Spin relaxation time (**d**), Contact resistance to spin resistance ratio (**e**), Spin relaxation length (**f**) as a function of carrier density at 20 K.

Figure S5 shows the spin transport parameters for device C, with a mobility of 30,000 cm^{2}V^{-1}s^{-1} at 20 K. The spin parameters where extracted from the fitting of Hanle curves, as described in the main text. The range of values of the spin parameters P, τ_s and λ and their electron-hole symmetry are commensurate with the devices presented in the main text, indicating consistent behaviour of the device architecture.

Section 6. Exploring the mechanism of spin relaxation

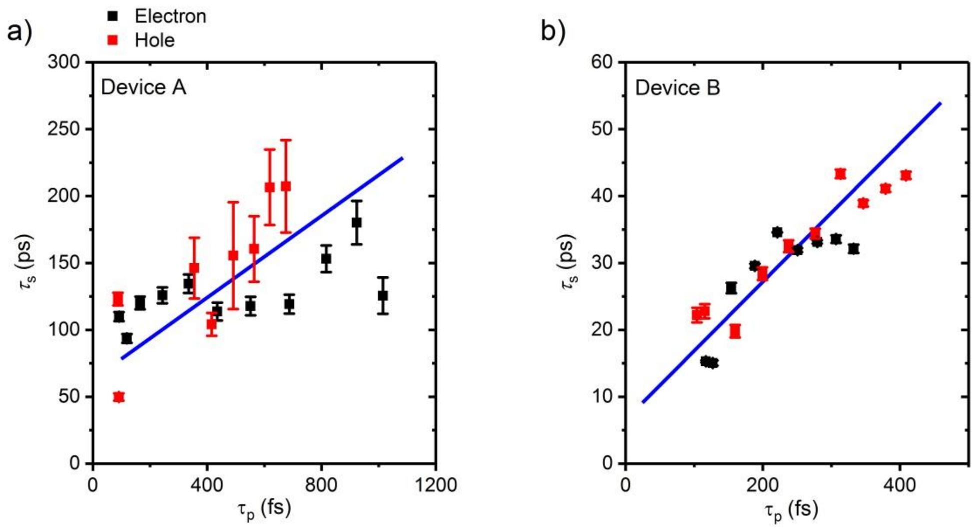

Figure S6. Scaling of spin and momentum relaxation times. **a, b,** A comparison between the spin- and momentum-relaxation times for devices A (**a**) and B (**b**). Black (red) squares are data from measurements made with the device in the electron (hole) regime. Blue lines are guides to the eye.

The momentum relaxation time is extracted from charge transport measurements, given by,

$$\tau_p = \frac{2D_C}{v_F{}^2}\,. \qquad (2)$$

Figure S6 shows the relation between the spin (τ_S) and momentum (τ_p) relaxation times for the devices A and B, discussed in the main text. This analysis shows larger momentum relaxation times are correlated with larger spin relaxation times, indicating an Elliot-Yafet[11,12] (EY)-like spin relaxation mechanism.

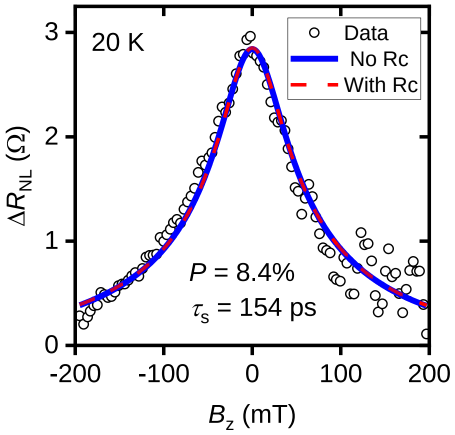

Figure S7. Role of contact resistance. Spin precession data, same as Fig. 3c, comparing a Hanle fit without considering contact resistance (blue line) and considering contact resistance (dashed red line).

The role of contact resistance on the extracted τ_S was assessed by performing fits to the Hanle data following the model by Fukuma et al.[13], which considers spin relaxation due to finite contact resistance (see Fig. S7). The fit including the contact resistance yields an extracted spin

lifetime just 10 ps higher, which is well within the fitting error. Such a fit takes into account the role of relaxation via a finite contact resistance, thus giving access to a longer lifetime associated with relaxation only within the channel. Analysis for our dataset showed that the difference is consistently small, below the error, in agreement with the nature of our non-invasive contacts.

Section 7. Long distance spin transport

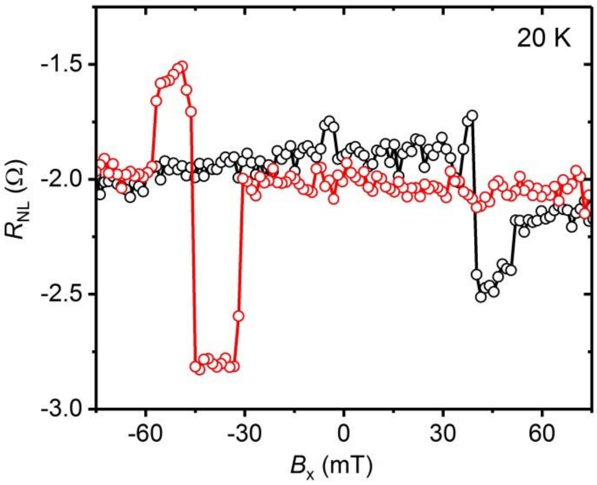

Figure S8. Long distance spin valve. Spin valve measurement at $n = -1.5\text{x}10^{12}$ cm^{-2} for a 12 µm long channel. The black (red) curve is for the up (down) sweep of the in-plane magnetic field.

Fig S8 shows the data from a spin valve measurement of a channel with a distance between injector and detector electrodes of L = 12 µm. Moreover, distributed along this length, there are five other 1D contacts across the channel. The observation of a spin-valve signal therefore confirms that our 1D magnetic contacts are of a non-invasive nature.

Furthermore, the observation of more than two switches (or more than two levels) in figure S8 indicates the detection of spin current by the closest reference contact to the detector, which is 3 µm further away. This implies spin transport for an even larger distance of 15 µm.

References.

1. Kretinin, A. V. *et al.* Electronic Properties of Graphene Encapsulated with Different Two-Dimensional Atomic Crystals. *Nano Lett.* **14**, 3270–3276 (2014).
2. Khestanova, E., Guinea, F., Fumagalli, L., Geim, A. K. & Grigorieva, I. V. Universal shape and pressure inside bubbles appearing in van der Waals heterostructures. *Nat. Commun.* **7**, 12587 (2016).

3. Auton, G., Kumar, R. K., Hill, E. & Song, A. Graphene Triangular Ballistic Rectifier: Fabrication and Characterisation. *J. Electron. Mater.* **46**, 3942–3948 (2017).
4. Levin, M. L. & Miller, M. A. Maxwell's 'Treatise on Electricity and Magnetism'. *Sov. Phys. Uspekhi* **24**, 904 (1981).
5. Goossens, A. (Stijn) M. *et al.* Gate-Defined Confinement in Bilayer Graphene-Hexagonal Boron Nitride Hybrid Devices. *Nano Lett.* **12**, 4656–4660 (2012).
6. Maassen, T., Vera-Marun, I. J., Guimarães, M. H. D. & van Wees, B. J. Contact-induced spin relaxation in Hanle spin precession measurements. *Phys. Rev. B* **86**, 235408 (2012).
7. Frisenda, R. *et al.* Recent progress in the assembly of nanodevices and van der Waals heterostructures by deterministic placement of 2D materials. *Chem. Soc. Rev.* **47**, 53–68 (2018).
8. Wang, L. *et al.* One-Dimensional Electrical Contact to a Two-Dimensional Material. *Science* **342**, 614–617 (2013).
9. Asshoff, P. U. *et al.* Magnetoresistance of vertical Co-graphene-NiFe junctions controlled by charge transfer and proximity-induced spin splitting in graphene. *2D Mater.* **4**, 031004 (2017).
10. Giovannetti, G. *et al.* Doping Graphene with Metal Contacts. *Phys. Rev. Lett.* **101**, 026803 (2008).
11. Elliott, R. J. Theory of the Effect of Spin-Orbit Coupling on Magnetic Resonance in Some Semiconductors. *Phys. Rev.* **96**, 266–279 (1954).
12. Yafet, Y. g Factors and spin-lattice relaxation of conduction electrons. *Solid State Phys.* **14**, 1–98 (1963).
13. Fukuma, Y. *et al.* Giant enhancement of spin accumulation and long-distance spin precession in metallic lateral spin valves. *Nat. Mater.* **10**, 527–531 (2011).
